# Complications Following Osseointegrated Transfemoral and Transtibial Implants: A Systematic Review

**DOI:** 10.7759/cureus.57045

**Published:** 2024-03-27

**Authors:** Christopher Rennie, Melissa Rodriguez, Katerina N Futch, Leighann C Krasney

**Affiliations:** 1 Medicine, Nova Southeastern University Dr. Kiran C. Patel College of Osteopathic Medicine, Clearwater, USA; 2 Medicine, Lincoln Memorial University-DeBusk College of Osteopathic Medicine, Harrogate, USA; 3 Orthopaedic Surgery, California Pacific Orthopaedics, San Francisco, USA

**Keywords:** transcutaneous limb osseointegration, implant osseointegration, post-operative complications, amputation, prosthetics

## Abstract

Lower limb amputation is a common orthopedic surgery in the United States and can be performed either above or below the knee. Prosthetics are typically externally fitted to the patient’s residual stump; however, osseointegrated implants offer a potential alternative to this process. Transcutaneous limb osseointegration involves the intramedullary anchoring of an implant that can later attach to a prosthetic via a stoma in the residual limb. There are proposed benefits to this, including decreased skin and soft tissue complications as well as an increased sense of stability. As this is a relatively new procedure, the complications and efficacy are not well supported by the literature at this time. The primary aim of this analysis was to synthesize the currently available data on transfemoral and transtibial osseointegration in order to improve our understanding of the potential complications of the procedure. A literature search was performed in the following databases: Biomedical Reference Collection, CINAHL, Cochrane Library, and PubMed/MEDLINE. Articles were screened by three independent reviewers for studies written or available in English, study design, and study outcomes, including complications. No filter was applied for publication date, publication national origin, or sample size. A total of 20 articles were selected for the final qualitative analysis. This review demonstrates an overall low or non-inferior rate of both minor and severe complications in transtibial and transfemoral osseointegration. This procedure should be considered as an option during preoperative planning in the context of above-the-knee and below-the-knee amputations. However, continued studies with larger sample sizes and extended postoperative follow-up are necessary for a greater strength of recommendation.

## Introduction and background

Osseointegration as a surgical procedure has been in use since the mid-1960s, primarily within the fields of dentistry and audiology. After several decades of clinical trials, successful transcutaneous limb osseointegration for amputees was first performed in Sweden in 1990 [1]. However, it is still considered an innovative and rarely performed procedure in the United States, with FDA approval eventually secured in 2015 [1,2]. This potential alternative option for amputation and prosthetic selection is increasingly important, especially within the context of the 150,000 lower limb amputations performed each year in the United States alone [3].

In the extensive trials prior to and since its FDA approval, numerous studies have been conducted to examine several crucial facets of the osseointegration procedure. This included, but was not limited to, examining the implant material and surgical technique to optimize the current procedure, methods to extend the longevity of the implant, and ways to decrease the occurrence of complications. The current protocol for osseointegration requires amputation of the affected limb with preservation of neurovascular structures and subsequent implantation of a metal fixture within the patient’s residual bone. This implant is then left protruding through a stoma formed in the stump and later attached to a prosthetic leg for functional use [4]. While innovative, compared to traditional lower limb amputation, this procedure has proven to be more technically challenging [1].

Traditionally, patients with transfemoral or transtibial amputations would receive a socket-suspended prosthetic, which can be adjusted to specifically fit what remains of the patient’s limb. These types of prosthetics have an increase in patient-reported difficulties with above-the-knee amputations (AKA) compared to below-the-knee amputations (BKA), making AKA patients more likely to be wheelchair bound [5,6]. With the progression of medical technology, the osseointegrated implant (OI) was developed to help limit the complications that were seen with traditional socket-suspended implants, such as skin irritation, skin damage, improper implant fitting, chronic pain, and excessive sweating [7,8]. One prime example of OI use in the current literature references the benefits of osseointegration in athletes. In this population, OIs help avoid the complications of excessive sweating and skin irritation seen in traditional prosthetic systems, so as to not impede the patient’s athletic performance and quality of life [9].

Compared to osseointegration, traditional lower limb amputation is a well-studied and well-understood procedure. Current mainstay indications for lower extremity amputation in the United States include trauma, invasive malignancy, orthopedic hardware failure, necrotizing fasciitis, vascular compromise, and chronic nonhealing diabetic wounds [5,6]. These indications, in addition to the risks and benefits of the procedure, are readily available and discussed in the preoperative planning phase. On the other hand, both surgeons and patients alike may often be unaware of osseointegration as an alternative option. Current FDA approval (PMA P190009) includes the use of osseointegration, typically as a secondary procedure following the failure of a socket-suspended prosthetic; however, it may also be utilized as a primary option in patients expected to not tolerate traditional socket-suspended systems [2]. Considering the overall novelty of transcutaneous limb osseointegration, there is an even greater disparity in the literature on postoperative complications. One major concern that may influence preoperative selection is the potential for infection, primarily due to the creation of a stoma and an iatrogenically made communication from the environment to the residual bone [10]. The complications involved in this procedure are not well understood by current data, and thus the purpose of this paper is to review the literature and provide greater insight into the expected complications encountered with transfemoral and transtibial OIs.

## Review

Methods

Article Eligibility Criteria

**Figure 1:**
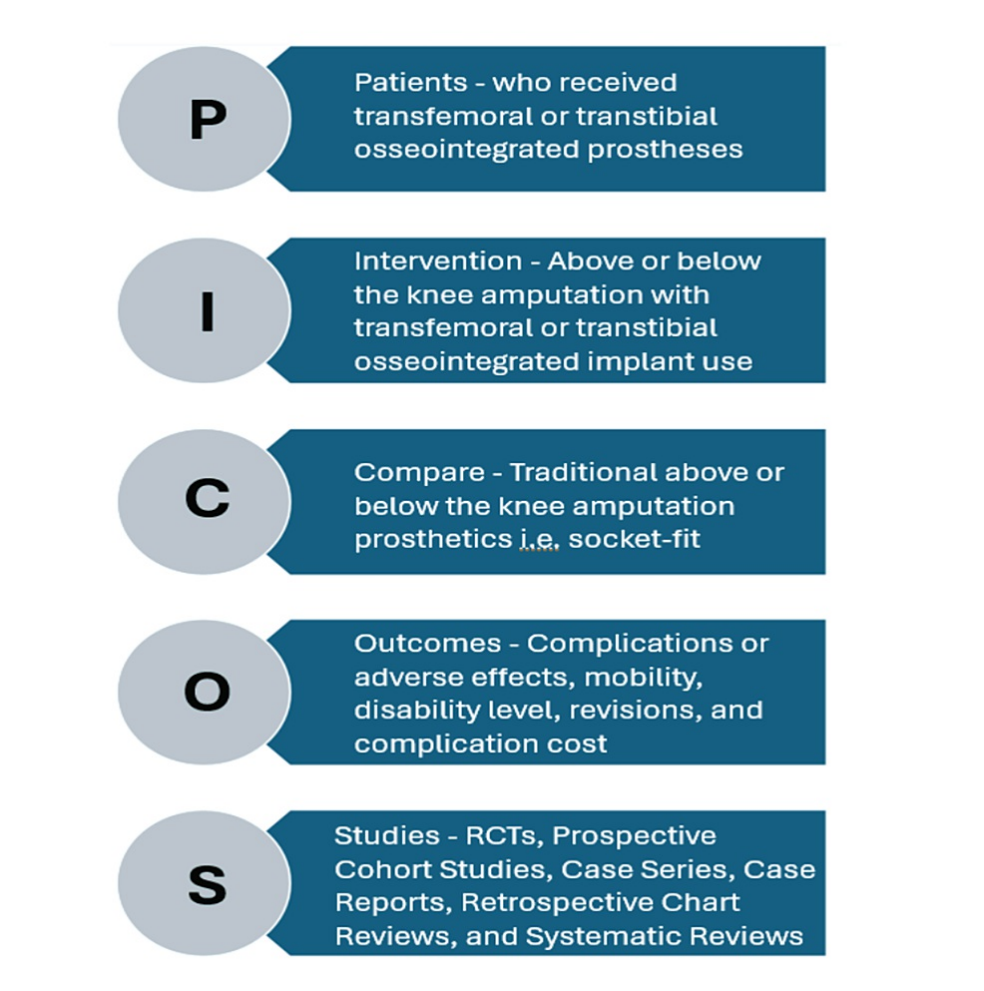
PICOS framework for article characteristic eligibility PICOS, Patients, Intervention, Compare, Outcomes, and Studies; RCT, randomized controlled trial Image credit: Christopher Rennie

The inclusion of articles for this review was based on content regarding transfemoral or transtibial placement of osseointegrated prostheses and subsequent complications evaluated. As seen in Figure 1, this review utilized the Patients, Intervention, Compare, Outcomes, and Studies (PICOS) format of article characteristic eligibility.

Search Strategy and Terminology

The literature search in this review was performed utilizing the following databases: Biomedical Reference Collection, CINAHL, Cochrane Library, and PubMed/MEDLINE.

The keywords and Boolean operators used for this review include the following: “osseointegration” OR “osseointegration limb replacement” OR “osseous prosthesis” OR “osseointegrated prosthesis” AND “complications” OR “adverse effects” OR “fracture” OR “infection” AND “amputation” OR “BKA” OR “AKA” OR “knee” OR “tibial” OR “femoral.”

The initial literature search was performed in September 2023, utilizing the PICOS eligibility criteria above. Additionally, only articles with full access and published or available in English were included.

Article Screening and Selection

This review was conducted in accordance with the Preferred Reporting Items for Systematic Reviews and Meta-Analyses (PRISMA) guidelines. Three reviewers independently screened the articles generated by the search terms in the listed databases. Articles were screened in the order of duplication removal, title relevance, abstract content, and full article content. If an article failed to meet inclusion criteria at any step in this process, it was excluded from the final article selection. After blind review, if any discrepancies in article inclusion were noted, these were discussed and agreed upon prior to qualitative analysis. Additionally, the methodological quality of studies was assessed independently by each reviewer, and the risk of bias was scored for each study prior to inclusion.

Data Extraction and Analysis

Selected articles were reviewed, and data was extracted from each regarding publication information, patient sample and characteristics, complications evaluated, primary outcomes of each study, and the overall recommendation with regard to osseointegration. This data was recorded in Table 1 and utilized for qualitative analysis.

**Table 1:** Summary of data extracted from included studies OI, osseointegrated implant; PRO, patient-reported outcome; RCT, randomized controlled trial; SIRS, systemic inflammatory response syndrome

Study	Year	Country	Study design	Patients	Complications evaluated	Main results	Stance on osseointegration
Banducci et al. [11]	2023	Australia	Systematic review	N = 803 (19 studies: 4 one-stage, 14 two-stage, and 1 one- and two-stage)	Superficial infection (one-stage: 38% vs two-stage: 52%), osteomyelitis (one-stage: 0% vs two-stage: 10%), implant failure (one-stage: 1% vs two-stage: 9%), and fracture (one-stage: 13% vs two-stage: 12%)	The one-stage approach is favorable compared to the two-stage	Favorable (one-stage)
Matthews et al. [12]	2019	United Kingdom	RCT	N = 18	Implant removal (5 implants, 28%), deep infection (3 implants, 17%), peri-implant infection (2 implants, 11%), superficial infections (11 implants, 61%), and chronic pain (1 implant, 5.6%)	Osseointegrated prostheses are associated with complicated infections but overall improve patient quality of life and are a reliable option	Favorable
Hagberg et al. [13]	2023	Sweden	Nonrandomized prospective cohort study	N = 51	Implant removal (N = 8; 15.69%), mechanical complications (rate: 3.9 per 10 person-years), and deep infection (N = 16; 31.37%)	PROs improved in prosthetic use, mobility, problems, and global score, but mechanical complications remain a concern. Despite this, the study questions the long-term sustainability and increased cost risk of an osseointegration prosthesis	Favorable
Black et al. [14]	2022	United States	Retrospective chart review	N = 25	Complication rates per year and costs: soft tissue infection (29%, $435), bone/implant infection (11%, $11,721), neuroma development (14%, $14,659), and mechanical failure (17%, $46,513)	The osseointegrated prosthesis was favored in terms of cost-effectiveness. It provides a higher quality of life with less expense	Favorable
Davis-Wilson et al. [15]	2023	United States	Nonrandomized prospective cohort study	N = 12	Increased disability (N = 2; 16.67%), decreased mobility (N = 1; 8.33%), and decreased activity level (N = 2; 16.67%)	OIs overall reduced disability, improved mobility, and improved balance confidence	Favorable
Black et al. [16]	2023	United States	Retrospective chart review	N = 60	Soft tissue infections (N = 25; 41.67%): osteomyelitis (N = 5), symptomatic neuromas (N = 6), and soft tissue revisions (N = 7)	Postoperative complications include soft tissue infections, and risk factors can be modifiable (BMI and center experience) or unmodifiable (sex and age).	Neutral (more research is needed)
Khemka et al. [17]	2015	Australia	Retrospective chart review	N = 4	Superficial infection (N = 1; 25%)	All patients improved physical and mental function, ambulation, and activity levels, but there were no statistically significant differences	Favorable
Hoellwarth et al. [18]	2022	United States	Retrospective chart review	N = 485	Death (N = 19; 3.9%): unrelated to osseointegration (N = 17; 3.5%); directly related to osseointegration (N = 2; 0.4%)	Osseointegration is a relatively safe procedure with a low risk of mortality	Favorable
Haket et al. [19]	2016	Germany	Case-control study	N = 27	No complications were recorded aside from increased cortical thickening	Transfemoral OIs increase periprosthetic cortical thickness in the first two years (most changes occurred postoperatively in the distal medial zone)	Favorable
Akhtar et al. [20]	2022	United Kingdom, United States, and Australia	Case series	N = 10	Complication rates: debridement (1 per patient), refashioning (1.3 per patient), periprosthetic fracture and implant removal (N = 1; 10%), needed one additional surgery (N = 7; 70%), and needed multiple additional surgeries (N = 5; 50%)	Better mobility is noted for patients who receive an osseointegration prosthesis following an infected TKR. Seven out of nine patients were not able to walk preoperatively, and eight out of nine alive patients showed slight to significant improvement in six-minute walking tests postoperatively	Favorable
Aschoff and Juhnke [21]	2016	Germany	Retrospective chart review	N = 86	Intramedullary infections (N = 3; 3.48%), aseptic loosening (N = 2; 2.32%), chronic soft tissue infections (N = 2; 2.32%), implant failure (N = 1; 1.16%), peritrochanteric or lateral femoral neck fractures (N = 6; 6.98%), and periprosthetic fracture (N = 1; 1.16%)	Gait security and symmetry improved. Soft-tissue infections can be prevented. High patient satisfaction	Favorable
Groundland et al. [22]	2022	USA and Canada	Retrospective chart review	N = 20	Spindle fracture and loosening (N = 3; 15%), revision surgery without implant removal (N = 11; 55%), and infection requiring revision (N = 4; 20%)	Complications seen after the after the post-osseointegration procedure occur early after surgery. No complications occurred after 29 months of surgery. There is no radiographic evidence of cortical atrophy or stress shielding. Overall, this is a safe option once the immediate postoperative period has been successful	Favorable
Kagan et al. [23]	2017	United States	Retrospective chart review	N = 116	Mechanical and overall failure are higher in the distal femur and transtibial implantation than in the proximal femur. Overall aseptic mechanical failure rate: 7%; overall failure rate: 25%	This study recommends proximal transfemoral osseointegration. There are possible advantages in decreasing aseptic failure, preventing stress shielding, allowing short-segment fixation, and preserving bone stock	Favorable (proximal transfemoral)
Reif et al. [24]	2021	United States	Retrospective chart review	N = 31 (18 transfemoral reconstructions and 13 transtibial reconstructions)	Bacterial infections (N = 6; 19.35%), surgical debridement of distal bone (N = 1; 3.23%), septic loosening (N = 1; 3.23%), aseptic loosening (N = 1; 3.23%), displaced proximal femoral fractures (N = 2; 6.46%), and soft tissue impingement (N = 1; 3.23%)	Significant improvement in walking post-osseointegration. Six patients were unable to use a prosthesis before surgery, and all were able to use a prosthesis and walk after surgery. There was a significant improvement in pain	Favorable
Wood et al. [25]	2020	United Kingdom	Retrospective chart review	N = 7	Postoperative SIRS (N = 6; 85.71%) and femoral fractures (N = 3; 42.86%)	Transfemoral osseointegration was successful even in a patient population with significantly complex injuries, such as veterans. Pain is an important complication that must be addressed when utilizing this form of prosthetic	Favorable
Atallah et al. [26]	2020	Netherlands	Retrospective chart review	N = 91	Soft tissue infections (N = 21; 23.08%) and septic implant failure (N = 1; 1.10%)	Most of the complications encountered were relatively easy to treat, which suggests that osseointegration is a safe procedure	Favorable
Hoellwarth et al. [27]	2020	Australia, United Kingdom, and Iraq	Retrospective chart review	N = 458	Periprosthetic fracture (N = 22; 6.3%) and inadequate fixation and additional surgery (N = 2; 0.57%)	There was a 6.3% risk of fracture for each kg above 80.4 kg. There was a 4.2% rate of fracture in the cohort. Women are more at risk for fractures after falling. Although there is a risk of fracture, it is a recommended procedure. In addition, patients maintained better mobility after the procedure	Favorable
Goldman et al. [28]	2016	United States	Retrospective chart review	N = 79	Death (N = 4; 5.06%), implant failure (N = 21; 26.58%), infection (N = 10; 12.66%), soft tissue failure/arthrofibrosis (N = 4; 5.06%), aseptic loosening (N = 2; 2.53%), and need revision surgery (N = 36; 45.57%): mechanical (N = 21; 26.58%) and nonmechanical (N = 15; 18.99%)	Osseointegration is a relatively safe procedure with a survival rate of 81% (at five and 10 years). Most of the injuries occurred within the first two years post-op via a twisting mechanism of injury	Favorable
Monument et al. [29]	2015	United States	Retrospective chart review	N = 18	Infection (N = 2; 11.11%), arthrofibrosis (N = 1; 5.56%), local disease recurrence (N = 1; 5.56%), and mechanical (aseptic) failure (N = 2; 11.11%)	The mechanical failures encountered were within the first 30 months post-op. The overall survivorship rate for post-op failure was 12 out of 18	Favorable
O’Donnell [30]	2009	United States	Retrospective chart review	N = 16	Aseptic loosening (N = 1; 6.25%), deep infection (N = 3; 18.75%), periprosthetic fracture (N = 2; 12.5%), metastatic disease (N = 4; 25%), and death (N = 2; 12.5%)	Transtibial reconstruction is more difficult and risky due to the proximity of the neurovasculature and the limited surface area. More complications are expected in tibial reconstruction due to the precision needed in the limited space to avoid adverse reactions postoperatively	Favorable (transfemoral)

Results

**Figure 2:**
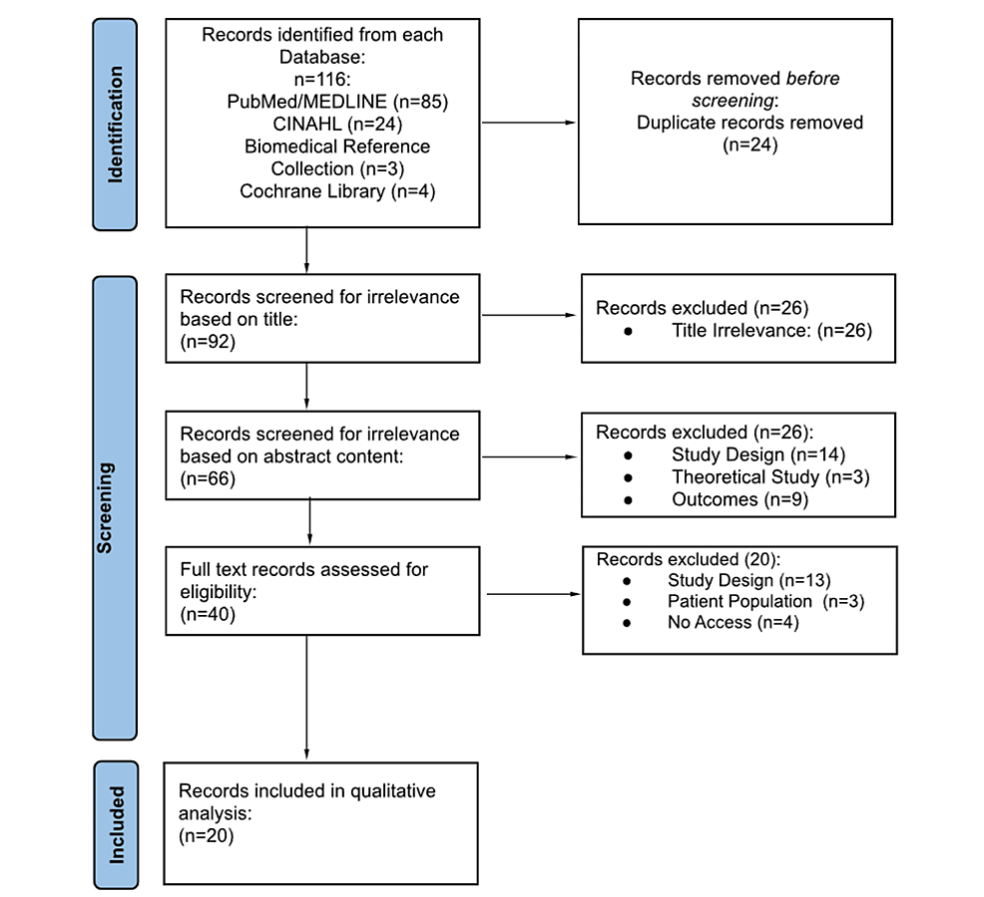
PRISMA flow diagram: inclusion and exclusion of articles from database search PRISMA, Preferred Reporting Items for Systematic Reviews and Meta-Analyses

The initial search query across all databases yielded a total of 116 articles. Details regarding subsequent article exclusion can be seen in Figure 2, with a final total of 20 articles included for qualitative analysis. Extracted data from each article regarding publication information, patient sample characteristics, complications, main results, and stance on osseointegration are recorded in Table 1.

All studies included in the qualitative analysis were performed in the following countries: United States (N = 9, 45%), United Kingdom (N = 2, 10%), Australia (N = 2, 10%), Germany (N = 2, 10%), Netherlands (N = 1, 5%), Sweden (N = 1, 5%), and multinational (N = 3, 15%). Multinational studies included the United States, the United Kingdom, Australia, Canada, and Iraq. Overall, studies from nine individual countries were included in this review. As no filter was placed on the publication date, this review included a comprehensive set of studies from 2009-2023 [11-30].

Across the 20 eligible studies, a total of 2,417 patients were analyzed, with patient sample sizes ranging from 4 to 803, the mean being 120.9 patients. Further patient information and demographics such as age, sex, gender, race, and ethnicity were not reported in all studies and thus were not included as a statistical measure. Study designs included retrospective chart reviews (N = 14, 70%), case series/case-control studies (N = 2, 10%), nonrandomized prospective studies (N = 2, 10%), systematic reviews (N = 1, 5%), and randomized controlled trials (N = 1, 5%) [11-30].

With regard to complications, 95% of studies (N = 19) reported at least some level of complications in the patients analyzed [11-18,20-30]. Of the complications noted, the most commonly mentioned was infection, with 65% of studies (N = 13) reporting any form of infection. Infection types included superficial or stoma infections, deep infections or osteomyelitis, and peri-implant infections. A total of 313 patients (12.95%) across the 20 studies were reported to have a superficial, stoma, or unspecified bacterial infection. A total of 75 patients (3.10%) reportedly suffered from deep infections or osteomyelitis, and three patients (0.124%) experienced peri-implant infections [11-14,16,17,21,22,24,26,28-30].

The following additional complications were recorded from the 19 studies and 2,417 patients: fractures (N = 80, 3.31%) [11,20,21,24,25,27,30], implant failure/removal (N = 72, 2.98%) [11-13,21,26,28], need for revision surgery (N = 65, 2.69%) [16,22,28], surgical debridement (N = 11, 0.455%) [20,24], septic or aseptic implant loosening (N = 10, 0.414%) [21,22,24,28,30], soft tissue neuromas (N = 9, 0.372%) [14,16], postoperative systemic inflammatory response syndrome (SIRS) (N = 6, 0.248%) [25], increased disability/decreased activity/decreased mobility (N = 5, 0.207%) [15], soft tissue arthrofibrosis (N = 5, 0.207%) [28,29], metastatic disease (N = 4, 0.165%) [30], chronic pain (N = 1, 0.041%), soft tissue impingement, (N = 1, 0.041%) [24], local disease recurrence (N = 1, 0.041%) [29], and death (N = 25, 1.03%) [18,28,30]. Of the deaths listed, 8.00% (N = 2) were directly related to osseointegration [18].

Overall, 95% (N = 19) of the studies determined that osseointegration was a favorable or reliable alternative for transtibial and transfemoral amputation and subsequent prosthetic planning [11-15,17-30]. Of these, three cited a preference for particular types of osseointegration, either a transfemoral or a one-stage approach [11,23,30]. One study (5%) maintained a neutral stance on osseointegration, citing the need for further research [16].

Discussion

Lower limb amputations have typically been performed with complete stump closure and subsequent socket-suspended prosthetic fitting [5,6]. Although a newer procedure, osseointegration offers an alternative option to this traditional process, with the placement of an intramedullary metal rod, the formation of a stoma, and the eventual direct anchoring of a prosthetic device [4]. The purpose of this review was to provide a qualitative analysis of the literature surrounding OIs in patients who underwent AKAs and BKAs, with the primary goal of highlighting expected complications and their overall efficacy.

The results of this review exhibit a strong recommendation for the use of OIs for lower limb amputees. Of the 20 eligible studies included, 19 (95%) provided a positive or favorable outlook on the use of transcutaneous limb osseointegration, with the remaining one (5%) maintaining a neutral stance [11-30]. Although the procedure is more technically challenging, osseointegration appears to be an efficacious procedure with favorable patient-reported outcomes (PROs) and a relatively low risk [1]. In addition to a low rate of overall complications, the results demonstrate even lower rates of severe complications, with no adverse effect aside from infection holding an incidence over 3.31%.

It is important to note that these rates were not solely limited to patients who were otherwise healthy. Certain severe and rare complications, such as metastatic disease spread, local disease recurrence, and death, were found in studies whose patients had several serious preexisting comorbidities [18,29,30]. With this context in mind, the true incidence of complications may actually be lower than what is represented here, further adding to the strength of OIs in healthy patients.

Complications Observed in Osseointegration

The following 16 types of complications were recorded from the 20 studies included in this qualitative analysis: infection, fractures, implant failure or removal, revision surgery, surgical debridement, implant loosening, soft tissue neuroma formation, postoperative SIRS, decreased activity and mobility, soft tissue arthrofibrosis, metastatic disease occurrence, local disease recurrence, soft tissue impingement, chronic pain, and death.

Of these, the most commonly reported complication was infection, with an incidence of 16.18% (N = 391) across all patients. While the infection rate with osseointegration is just under one out of every six patients, this is not an alarming finding in the context of surgical procedures, especially given their complexity. Invasive procedures such as these typically have high rates of infection. However, 16.18% is considered quite low comparatively. Current literature shows that traditional lower limb amputations with stump closure have infection rates upward of 40% [31]. In a study by Coulston et al., they found patients undergoing BKAs to be at a significant risk, with rates as high as 82.9%. Patients in the same study who were treated with AKAs were also found to have a high infection risk, with a rate of 19.6% [32].

As previously stated, aside from infection, no other complication had an incidence greater than 3.4%. Fractures associated with OIs carried the next highest rate of occurrence, affecting 3.31% of patients in this analysis. Compared to a traditional amputation and a socket-suspended prosthesis, this may be a potential drawback in the use of OIs. Literature suggests that patients with socket-suspended prosthetics have a 2.2% risk of fracture over five years, a rate slightly lower than found in the group of patients in this analysis [33]. Despite this, there are still active developments in the process of implant material and sizing, with the aim of decreasing this complication over time [1].

Implant failure/removal and revision surgery are two major complications found in this analysis, although the incidence for each is relatively low at 2.98% and 2.69%, respectively. With regard to surgical revision, current literature suggests roughly 25% of lower limb amputees undergo some form of revision [34,35]. Comparatively, patients who underwent transcutaneous limb osseointegration in the included studies were much less likely to require surgical revision. It is important to note that many of these studies are relatively recent, and patients may have sought revisions after study completion or publication. Implant removal is a difficult metric to compare, as traditional lower extremity amputees do not have implants. In the field of orthopedics in general, there is varying data on rates of implant and hardware removal, ranging from around 10-50% depending on the type of surgical fixation [36,37].

A major advantage OIs inherently hold over socket-suspended prosthetics is patient satisfaction surrounding soft tissue complications. The overall incidence of non-infection-related soft tissue complaints in patients with OIs, including neuroma formation, impingement, and arthrofibrosis, was 0.62% (N = 15) [14,16,24,28,29]. One study found nearly 75% of patients with socket-suspended prostheses experienced skin breakdown, pressure sore development, irritation, excessive sweating, and overall discomfort [38]. OIs provide an excellent alternative in the context of skin protection, primarily due to their inherent design. The implant is anchored directly within the bone through a stoma, whereas socket-suspended prosthetics apply constant contact, friction, and pressure to the residual limb stump itself [5,6].

Strengths, Limitations, and Future Direction

This review holds several inherent strengths in providing a strong recommendation for transcutaneous limb osseointegration in the setting of AKAs and BKAs. The literature search utilized in this qualitative analysis investigated four high-quality databases that encompassed a vast majority of the available peer-reviewed scholarly work. Additionally, the selected query terms and Boolean operators used for this search provided a large subset of articles for further analysis. Inclusion criteria with no limits on publication date, national origin, sample size, and types of complications also allowed for a comprehensive set of articles to be assessed. In the evaluation of these studies, three separate researchers performed independent blind reviews and concurrent quality analyses, further strengthening the validity of this literature review.

With any study, there are limitations to keep in mind. As seen by the search query results, there is an overall scarcity of literature surrounding this topic, which makes it more difficult to produce a generalizable recommendation. Beyond this, a majority of the articles included in this analysis were retrospective chart reviews, with a limited number of high-quality randomized studies. Additionally, patient demographics and specific case information are important parameters this review was unable to investigate, as not all studies recorded these variables. Some of the case details that would aid in building a stronger recommendation include follow-up adherence, postoperative rehabilitation participation, and implant size and material, as each of these may significantly affect healing and subsequent complications.

Future research is needed to facilitate a greater understanding of transcutaneous limb osseointegration, especially in the context of complications and functional outcomes. Further studies on topics such as PRO scores, the interplay of patient demographics, various joints of interest, comparative efficacy of amputation and prosthetic methods, and large-sample randomized trials will help to create a more definitive recommendation that both patients and surgeons can rely upon.

## Conclusions

Transcutaneous limb osseointegration is still a relatively new procedure within the last three decades, with limited data and literature available on its effectiveness. While traditional amputation methods and subsequent external prosthetic fitting remain the standard practice, osseointegration is an option that is oftentimes unexplored. The aim of this study was to synthesize the currently available literature and understand the risks, benefits, and overall perception of transtibial and transfemoral limb osseointegration. We illustrate the relative efficacy of osseointegration in the context of low total complication rates and a low frequency of severe complications and revisions. Ultimately, the primary outcomes of this study were successfully analyzed, showing transcutaneous limb osseointegration for AKAs and BKAs is an efficacious option that orthopedic surgeons should educate patients on and consider as part of preoperative planning.
